# Quaternary diagnostics scheme for mucolipidosis II and detection of novel mutation in *GNPTAB* gene

**DOI:** 10.1186/s43141-021-00204-4

**Published:** 2021-08-03

**Authors:** Mona L. Essawi, Ekram M. Fateen, Hanan A. Atia, Noura R. Eissa, Eman H. Aboul-Ezz, Mona M. Ibrahim, Heba A. Hassan, Samia A. Temtamy

**Affiliations:** 1 Department of Medical Molecular Genetics, Division of Human Genetics and Genome Research, Dokki, Cairo, 12311 Egypt; 2 National Research Centre, Cairo, Egypt; 3 Department of Pharmacology and Toxicology, College of Pharmacy, Hail University, Hail, Kingdom of Saudi Arabia; 4 Department of Biochemistry, Faculty of Pharmacy (Girls), Al Azhar University, Cairo, Egypt; 5 Division of Oral & Dental Research, Department of Basic Dental Sciences, Dokki, Cairo, 12311 Egypt; 6 Division of Human Genetics and Genome Research, Department of Biochemical Genetics, Dokki, Cairo, 12311 Egypt; 7 Division of Human Genetics and Genome Research, Department of Clinical Genetics, Dokki, Cairo, 12311 Egypt

**Keywords:** Mucolipidosis, *GNPTAB gene*, NGS, ML II, Egyptian, Mutations

## Abstract

**Background:**

Mucolipidosis II (ML II α/β) is an inherited lysosomal storage disorder caused by deficiency of GlcNAc-phosphotransferase enzyme and results in mis-targeting of multiple lysosomal enzymes. Affected patients are characterized by skeletal deformities and developmental delay. Homozygous or compound heterozygous mutations in *GNPTAB* gene are associated with the clinical presentation. This is the first study to characterize the underlying genetics of ML among a cohort of Egyptian patients. ML II diagnosis established by clinical assessment, biochemical evaluation of enzymes, electron microscopy examination of gingival inclusion bodies, and molecular study of *GNPTAB* gene using targeted next-generation sequencing panel in 8 patients form 8 unrelated Egyptian families.

**Results:**

Sequencing revealed 3 mutations in *GNPTAB* gene; 1 novel frame-shift mutation in exon 19 (c.3488_3488delC) and 2 previously reported mutations (c.1759C>T in exon 13 and c.3503_3504delTC in exon 19). All patients were homozygous for their corresponding mutations and the parents were consanguineous.

**Conclusions:**

According to the established quaternary diagnostic scheme, ML II was the final diagnosis in eight patients. The most common mutation was the frame shift c.3503_3504delTC mutation, found in 5 patients and associated with a severe phenotype.

## Background

Mucolipidosis types II (ML II α/β) is an inherited lysosomal storage disorder known as I-cell disease (OMIM# 252500). ML II α/β is caused by deficiency of the uridine diphosphate (UDP)-N-acetylglucosamine: lysosomal enzyme N-acetylglucosamine-1-phosphotransferase (GlcNAc-phosphotransferase; EC 2.7.8.17) [12, 16]. This membrane-bound enzyme is responsible for the initial synthesis step of Mannose-6-Phosphate (M6P). Without M6P, newly synthesized lysosomal hydrolases processing and packaging are impaired and are misdirected and secreted into extracellular spaces. An attenuated form of the disease was also reported as mucolipidosis type III (ML III). ML III is classified into 2 subtypes: ML III α/β (OMIM# 252600, also known as Pseudo-Hurler polysdystrophy) and ML III γ (OMIM# 252605) [27, 29].

They are rare genetically related inherited disorders of lysosomal metabolism. Their combined frequency is about 1:422,000 live births. Worldwide estimates showed that ML II occurs in about 1 in 100,000 to 400,000 individuals [7]. The estimated incidence of ML is 2.5 and 10 cases per 1,000,000 live births across the world [15, 17]. In the young founder population of the north-eastern region of Quebec [Saguenay–Lac-Saint-Jean (SLSJ)], ML II prevalence reached of 1/6184 at birth with an estimated carrier rate of 1/39 [18].

ML II α/β represents the most severe form of the disease. Clinical symptoms of ML II α/β usually start at birth and sometimes it is evident prenatally; the disease course is very aggressive. ML II α/β patients are characterized by coarse facial features, dysostosis multiplex—like deformities, hypertrophic gingiva, and delayed psychomotor development. Cardiopulmonary complications are the principal cause of death during the first decade of life in ML II α/β patients [3, 11, 19, 21, 28].

The GlcNAc-phosphotransferase enzyme is a hexameric complex (2 α, 2 β, and 2 γ subunits) encoded by 2 genes. The α and β subunits are encoded by *GNPTAB* gene (MIM: 607840) which contains 21 exons and span a genomic region of 85 kb on chromosome 12q23.3. The protein encoded has 1256 amino acids in length with 144 kDa molecular mass for α/β precursor. Mutations in this gene were found to be responsible for ML II α/β and a more attenuated form; ML III α/β. While mutations in the *GNPTG* gene (MIM: 607838) at chromosome 16p13.3, which encodes the γ subunit were associated with ML III γ [27].

Almost 25% of the known *GNPTAB* mutations are located in the 1112 bp exon 13. The majority of the mutations (72%) were found in individual families; only 25 GNPTAB mutations (10%) were identified in more than two families. The most prevalent *GNPTAB* mutation c.3503_3504del was identified in 131 individuals and does not cluster geographically [26].

The study aimed to identify for the first time the molecular pattern of ML II among clinically suspected patients in order to confirm the diagnosis by molecular analysis and to establish a four-stage diagnostic scheme including clinical evaluation, electron microscopy examination, biochemical, and molecular studies. This would provide the more accurate diagnosis and prognosis, prenatal diagnosis, and family counseling.

## Method

### Ethics approval and consent to participate

The Medical Research Ethics Committee at the host research institute approved the study protocol and results. Written informed consents were obtained from legal guardians of the participants according to guidelines of the Medical Research Ethics Committee.

### Patients

Eight patients from eight unrelated families were referred from the Limb Malformations & Skeletal Dysplasia Clinic (LMSDC) and Biochemical Genetics Department. The patients were 5 males and 3 females of age ranged from 3 months to almost 4 years. Two amniotic fluid samples obtained from pregnant mothers of two unrelated patients included in the study were subjected to molecular analysis. Parents’ consanguinity was confirmed in all studied families.

### Methods

#### Lysosomal enzyme analysis

The β-Hexosaminidase-A enzyme activity was measured in plasma isolated from fresh peripheral blood samples using fluorogenic substrate 4-methylumbelliferyl-6-sulfo-2-acetamido-2-deoxy-β-d-glucopyranoside. The assay was performed according to the protocol proposed by [13]. The reference range for this enzyme was 50–200 μmol/l/h.

#### Electron microscopy examination

Oro-dental examination started with clinical evaluation of the mouth. Gingival biopsy was then examined by bright field illumination in Zeiss microscope using toluidine blue or methylene blue stain. Ultrathin section obtained from the selected area of the specimen was then stained with uranyl acetate and citrate and examined under the transmission electron microscope.

#### Molecular studies

For each participant, 3–5 ml of peripheral (venous) blood samples were withdrawn and transferred immediately to either PAXgene specific blood collecting tubes or polypropylene tubes containing 0.5 M EDTA (pH 8.0, violet cap) and mixed thoroughly to prevent clotting and to stop nuclease activity.

Genomic DNA was extracted from peripheral blood leukocytes. DNA concentration and purity were determined using NanoDrop® 2000 (Thermo Scientific, USA) and DeNovix® (DeNovix Inc. USA) to quantify the dsDNA.

The whole *GNPTAB gene* and the intervening sequences were sequenced by MiSeq® Sequencing System within a costumed panel of genes related to inherited genetic disorders. This panel was designed upon request by Illumina, USA. It consisted of 25 genes including *GNPTAB gene*. The library was prepared using the TruSeq® Custom Amplicon v1.5 Reagent kit (Illumina, USA). *GNPTAB* mutations detected were then confirmed by Sanger sequencing using ABI 3500 Genetic Analyzer (Applied Biosystems, USA) for both patient and their parents if available.

DNA was extracted from 20 cc amniotic fluid samples using a GeneJET Whole Blood Genomic DNA Purification Mini Kit (Thermo Scientific, USA) according to the manufacturer’s protocol, and then, Sanger sequencing was used to detect the mutation sites of the probands. Sequencing chromatograms visualized by Finch TV software and aligned with *GNPTAB gene* reference sequence (NM_024312.4) to identify nucleotide variations. ENTPRISE-X tool was used for in silico analysis of detected mutations.

This study is considered as case-series study, and such study type is more descriptive than being analytical so that no statistical analysis is needed.

## Results

All patients were provisionally diagnosed as mucolipidosis type II according to their clinical manifestations with dysostosis multiplex associated with biochemical increase in the extracellular activity of β-Hexosaminidase-A enzyme above reference range in accordance with specific electrophoretic separation pattern of urinary glycosaminoglycans (GAGs), and finally the identification of inclusion bodies in electron microscopy grids for the gingival biopsy. Only two patients deceased before completing their clinical assessment; however, ML was suspected according to the biochemical and electron microscopy (E.M.) findings.

Urinary GAGs were high for age with specific pattern in electrophoretic separation different from that of MPS.

### Clinical and oro-dental results

**Fig. 1:**
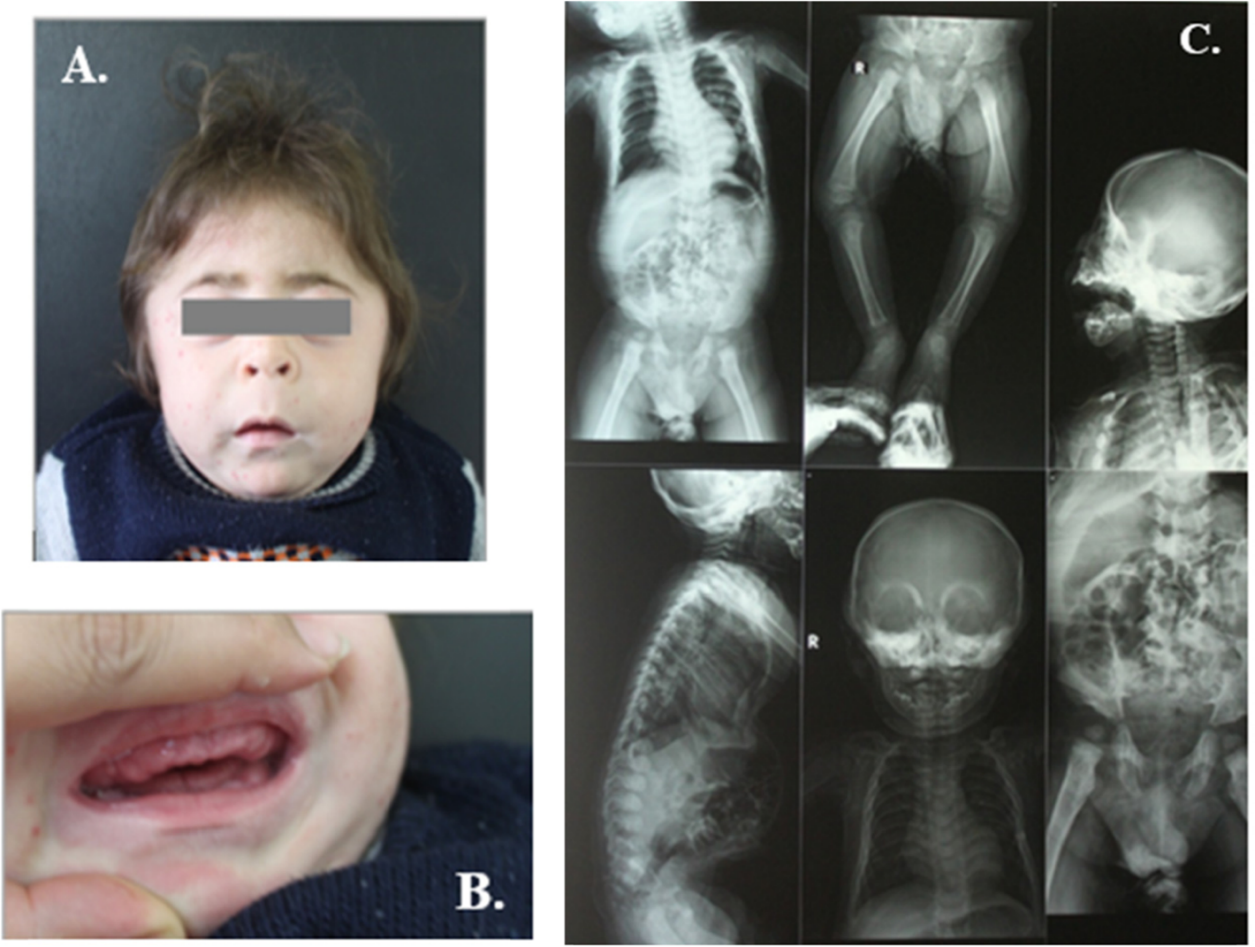
Patient no. 1 at the age of 9 months. **A** Clinical features of ML II including coarse facial features, long philtrum, tower skull, and short neck. **B** Hypertrophic gingiva of the mouth. C X-rays of patient no. 1 showing dysostosis multiplex at ends of long bones and vertebrae

**Fig. 2:**
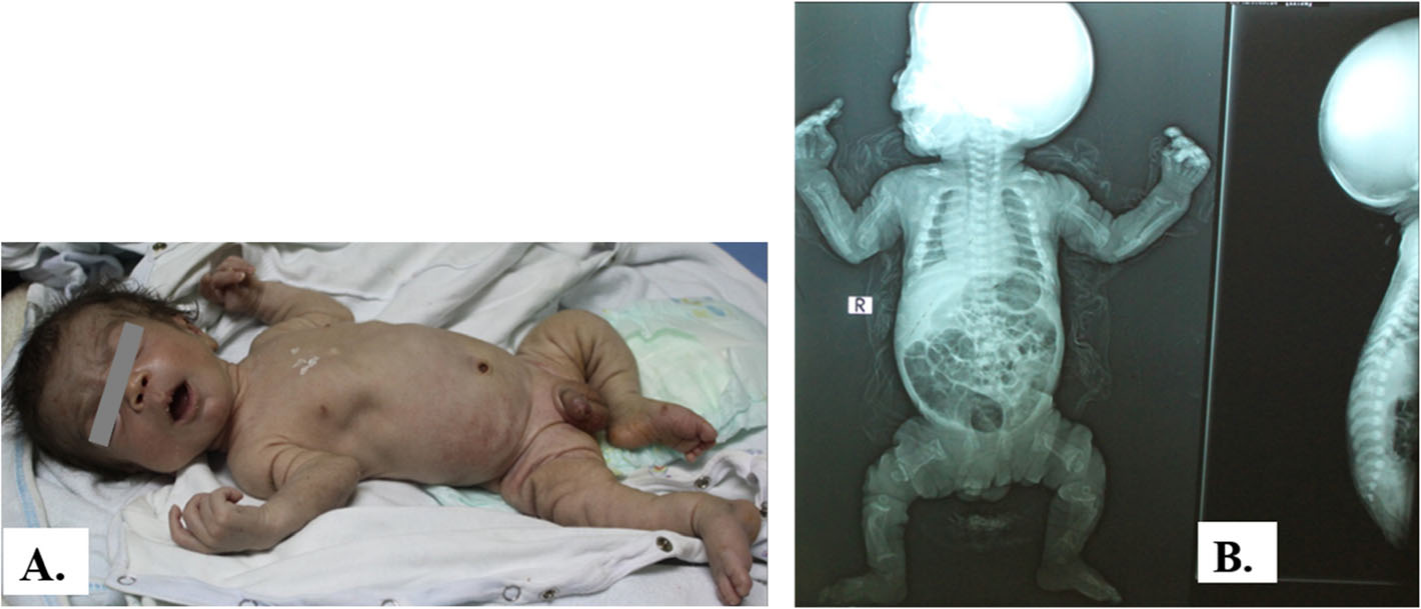
Patient no. 2 at the age of 3 months. **A** Coarse facies, digital contractures, indrawn chest wall, and Michelin tyre appearance of skin folds in the limbs. **B** X-rays showing short thick long bones with splayed metaphyses, small epiphyses, horizontal ribs and short, square or biconvex vertebral bodies

**Fig. 3:**
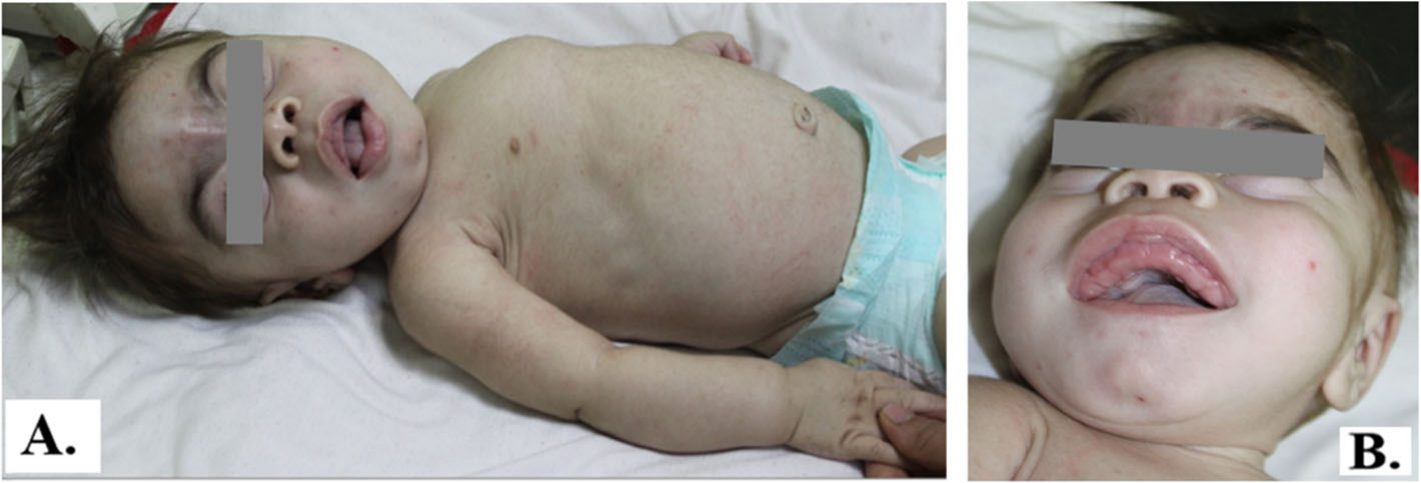
Patient no. 3 at the age of 1 year. **A** Clinical features of ML II including coarse facial features, pectus carinatum, and short neck. **B** Gingival hyperplasia

**Fig. 4:**
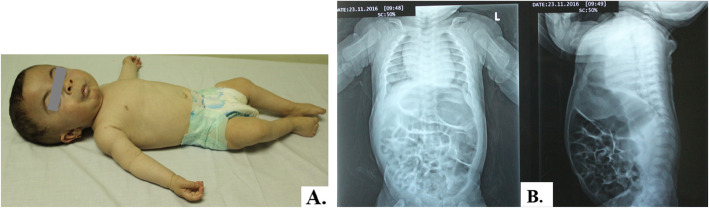
Patient no. 6 at the age of 1 year. **A** Skeletal X-rays showing short humerus, horizontal oar-shaped ribs. **B** Lateral X-ray spine showing short biconvex vertebral bodies and oar-shaped ribs. Note high square forehead, short upturned nares, chest deformity, and short limbs

The first signs and symptoms were noted either prenatally or at birth in most cases mainly in the form of coarse facies, delayed milestones, and progressive skeletal deformities (Figs. 1, 2, 3, and 4). Clinical data of 6 patients were summarized in Table 1. Certain clinical data of the remaining two patients (no. 5 and no. 8) were not available, as they deceased soon after referral.

**Table 1:** Clinical data of the 8 studied patients

Patient	Consanguinity	Short stature	Milestones	Coarse facies	Cardio-pulmonary complications	Gingival hypertrophy	E.M. Findings
1	+	+	Delayed	+	+	+	ML
2	+	+	Delayed	+	+	*NA*	*NA*
3	+	+	Delayed	+	+	+	ML
4	+	+	Delayed	+	+	+	ML
5	+	*NA*	Delayed	*+*	*NA*	+	ML
6	+	+	Delayed	+	−	+	ML
7	+	+	Delayed	+	+	+	ML
8	+	*NA*	Delayed	+	+	+	ML

*NA* not available

All patients were presented with dysostosis multiplex. Detailed skeletal survey has shown progressive abnormal skull shape in patient nos. 1, 3, and 6; oar-shaped ribs in patient nos. 3 and 4; bilateral lower limb bowing in patient nos. 6 and 7; in addition to kyphoscoliosis noted in patient nos. 3 and 6, and pectus carinatum in patient no. 7. Two patients had small head circumference, nos. 2 and 4. Mild brain atrophy could be elucidated from CT scans of patient nos. 6, 7, and 8. Cardiopulmonary complications including mitral valve prolapse, mitral valve regurgitation, atrial septal defect secundum, recurrent upper respiratory tract infections, dilated left ventricle, and pulmonary hypertension were evident in the echocardiography. Three families (nos. 1, 2, and 4) had a history of previously died sibling due to respiratory distress.

General oro-dental examination of the patients showed prominent long philtrum, thin lips, prominent premaxilla, retrognethia of maxilla, broad jaws with thick alveolar ridges, and gingival hyperplasia in all patients.

### Electron microscopy examination

**Fig. 5:**
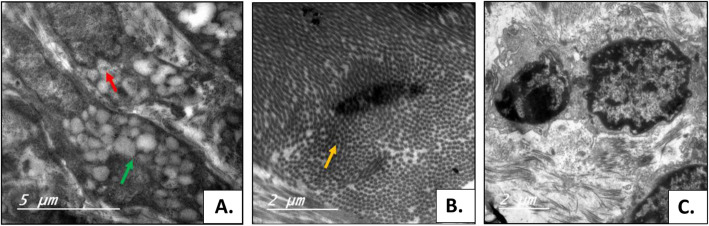
**A** Ultrastructure image showing intracellular lipid and mucoid vacuoles (denoted with green arrow) with nuclear invagination (denoted with red arrow). **B** Ultrastructure photograph showing increased number of collagen fibers. **C** Ultrastructure image showing nuclear invagination and two nucleoli

The ultrastructure examination with electron microscopy showed the characteristic picture of ML in seven patients with the presence of several inclusion bodies (Fig. 5A–C). Increased number of collagen fibers, widening of the intracellular spaces which are filled with lipid, inter- and intracellular large vacuoles of mucoid and lipid material varying in size with multiple nucleoli, and enlarged nucleus filling almost the cytoplasm with nuclear invagination.

### Lysosomal enzyme analysis

β-Hexosaminidase-A enzyme activity assay ranged from 263 to 702 nmol/l/h (495.5 ± 185) for 8 patients (Table 2). All the results were above the normal range of GNPTAB enzyme activity (n: 50–200 nmol/l/h.).

**Table 2:** Biochemical and molecular results in the 8 studied patients

Patient	Sex	Age of onset	Hex-A activity (μmol/l/h)	Gene	Exon	Mutation	Type
1	M	4 months	389.9	GNPTAB	13	p.R587X	Reported
2	M	Prenatally	*NA*	GNPTAB	13	p.R587X	Reported
3	M	At birth	672	GNPTAB	19	p.L1168QfsX5	Reported
4	F	At birth	651.1	GNPTAB	19	p.L1168QfsX5	Reported
5	M	*NA*	500.4	GNPTAB	19	p.L1168QfsX5	Reported
6	F	At birth	702	GNPTAB	19	p.L1168QfsX5	Reported
7	M	Prenatally	290	GNPTAB	19	p.L1168QfsX5	Reported
8	F	At birth	263	GNPTAB	19	p.T1163KfsX2	Novel

### Molecular results

#### NGS analysis of GNPTAB gene

Mutations of *GNPTAB gene* were detected in 8 unrelated patients. All patients were homozygous for their corresponding mutations (Table 2). Interpretation and bioinformatics analysis of the 8 different variants detected has shown that five of which were benign and three were pathogenic and disease causing. The five benign variants were 2 synonymous variants rs10778148 (c.1932A>G) and rs4764655 (c.18G>A), one intronic splice site SNV (c.3135+5T>C), one intronic SNV (c.3336-25T>C), and one intronic deletion (c.1613-25delA).

**Fig. 6:**
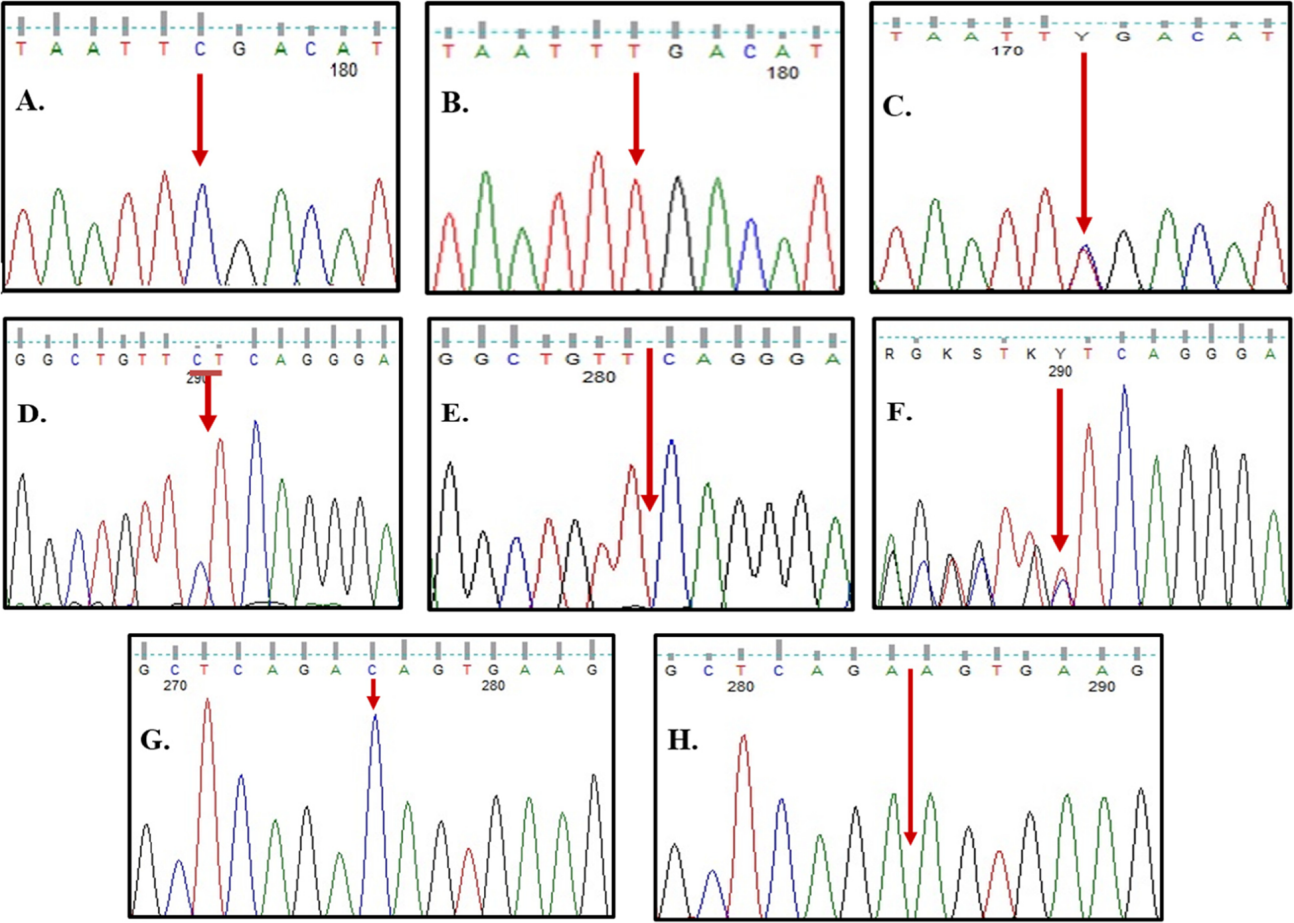
Sequence chromatograms of exons 13 and 19 of *GNPTAB gene*. **A** Wild type of exon 13. **B** Mutated sequence of exon 13 for patient nos. 1 and 2 (homozygous for p.R587X). **C** Heterozygous pattern for p.R587X mutation found in the parents. **D** Wild type of exon 19. **E** Mutated sequence of exon 19 for patient nos. 3–7 (homozygous for p.L1168QfsX5). **F** Heterozygous pattern for p.L1168QfsX5 mutation found in the parents. **G** Wild type of exon 19. **H** Mutated sequence of exon 19 for patient no. 8 (homozygous for p.T1163KfsX2)

However, the 3 pathogenic variants were a previously reported nonsense mutation in exon 13 c.1759C>T (p.R587X) that was detected in two patients. It caused the production of an inactive truncated precursor *GNPTAB* protein [25]. In both families, parents were heterozygous each for the corresponding mutation. The same mutation was also screened in two amniotic fluid samples (AF) one from each family. Wild-type sequences of exon 13 were detected in the AF sample from patient no. 1’s family. However, AF sample of patient no. 2’s family showed an affected sibling (Fig. 6A–C).

The second pathogenic variant was a frameshift deletion in exon 19, c.3503_3504delTC (p.L1168QfsX5), identified in 5 patients. It was previously reported as disease causing mutation for both ML II and Stuttering [9, 16]. It is resulted in frame shift of open reading frame (ORF) of the gene and premature stop codon is generated (Fig. 6D–F).

**Fig. 7:**
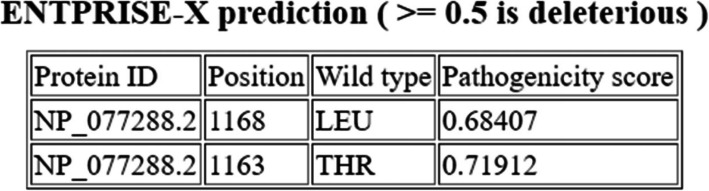
Enterprise-X tool report comparing the pathogenicity of the two frameshift mutations in exon 19 of *GNPTAB gene*; the reported p.L1168QfsX5 mutation and the novel single base deletion p.T1163KfsX2 (http://cssb2.biology.gatech.edu/entprise-x/)

The third pathogenic variant was a novel single base deletion (c.3488_3488delC) in exon 19, causing a frame shift mutation that changed threonine at position 1163 into lysine and led to a premature stop codon two amino acids away from the deletion site (p.T1163KfsX2) (Fig. 6G–H). Analysis with ENTPRISE-X was carried out; an algorithm for predicting human disease-associated frameshift and nonsense mutations. It scored the novel mutation 0.71912 which classifies it as a deleterious disease-causing or disease-damaging mutation (Fig. 7). Comparing the ENTRPRISE-X scores for both frameshift mutations, the novel mutation p.T1163KfsX2 scored higher than the reported p.L1168QfsX5 mutation (0.68407). This confirms the pathogenicity of the mutation.

### Sanger sequencing analysis

PCR amplification and sequencing were performed for exons 13 and 19 of *GNPTAB* gene to confirm the mutations detected by NGS.

The novel detected variant of *GNPTAB* gene in exon 19 was submitted to ClinVar database and gained accession number SCV000891127.1 (National Center for Biotechnology Information. ClinVar; Variation ID 623268, https://preview.ncbi.nlm.nih.gov/clinvar/variation/623268 (accessed April 22, 2019)).

## Discussion

Mucolipidosis type II (ML II) is an autosomal recessive lysosomal storage disorder with disturbed metabolism of many mucopolysaccharides or GAGs giving rise to clinical features similar to those encountered in patients with mucopolysaccharidoses (MPS). It resulted from impaired trafficking of lysosomal hydrolases to lysosomes due to total or near total deficiency of GlcNAc-phosphotransferase enzyme [3, 8, 12, 29].

Our study aimed mainly at establishing a diagnostic tool to confirm ML diagnosis in suspected patients. The diagnosis is confirmed by the detection of pathogenic variants (mutations) which affect the function of the GNPTAB gene product (protein), but we do not study polymorphic markers.

This study identifies the first *GNPTAB* mutations in eight Egyptian patients with ML II. The studied patients were selected based on three main inclusion criteria: their clinical presentations, oro-dental examination including E.M. picture of gingival biopsy, and β-Hexosaminindase-A enzyme activity assay. The whole 21 exons of *GNPTAB* gene were sequenced in 8 patients. Three different mutations were detected in exons 13 and 19; one of which was novel (p.T1163KfsX2 in exon 19).

Plante et al. linked the ML II phenotype observed in the probands to the deleterious effect of c.3503_3504delTC (p.L1168QfsX5) deletion mutation of the *GNPTAB* gene. It was described as a null allele, which means that protein coded (p.L1168QfsX5) do not produce any GlcNAc-phosphotransferase activity. The result of 2 nucleotide (CT) deletions from a repeating CTCT sequence may have caused a mismatch-repair error. The frameshift truncates the enzyme at amino acid 1171 in the β-subunits. Fibroblasts of patients homozygous for his mutation showed very low value of ≤ 1% GlcNAc-phosphotransferase activity. Thus, homozygous c.3503_3504delTC mutation is responsible for the phenotype presentation of MLII disease [14, 18].

In the majority of the studied populations, the (c.3503_3504delTC; p.L1168QfsX5) mutation of *GNPTAB* gene is the most frequent ML II causing mutation. It was the only detected mutation among 27 ML II obligatory carriers in French Canada [18]. In Italy and the USA, this mutation represented 51% (of 38 patients) and 22% (of 61 patients) of ML II/III α/β patients, respectively. In a study group of 21 ML II patients, Arabs, Turkish, and Irish origin of Israel and Palestinian Arabs, only 12 patients had p.L1168QfsX5 mutation [2, 14]. Also, it was detected in 51.1% of mutant alleles in homozygous pattern within a cohort of 14 ML II patients including 12 Italian, 1 Bangladeshi, and 1 Argentinian. In ML II Portuguese patients, it was the most frequent *GNPTAB* mutation found, accounting for 45% of the mutant alleles detected in the Portuguese series [6, 23]. It was also reported in Indian patients twice, in 2014 and in 2016 [1, 22].

Studies concluded that (c.3503_3504delTC; p.L1168QfsX5) could have a potential founder ancestor or molecular lesion. The origin and spread of the pathogenic c.3503_3504delTC variant was conducted through a worldwide haplotype diversity assessment including samples bearing the mutation. It inferred that they are descendants from a single founder ancestor probably in a peri-Mediterranean region out of Europe [4]. Nevertheless, this conclusion does not apply for the Chinese population. Where the majority of Chinese patients reported mutations were located in exon 13 or its intronic flanking regions [16].

Our results conformed c.3503_3504delTC mutation frequency to the previously published ones. The mutation was detected in 5 unrelated patients; 3 males and 2 females. It was found in homozygous pattern in more than half of our studied group (10/16 alleles). All of them were offspring of consanguineous marriages. Their symptoms mostly denoted at birth or shortly after. Four of the patients presented with typical ML symptoms including short stature, coarse facies, gingival hypertrophy, and variable degrees of brain affection.

The nonsense mutation c.1759C>T (p.R587X) was reported in two patients, nos. 1 and no. 2, both had severe manifestations of ML that were evident very early in life, and in one amniotic fluid sample for one of the families. The family history of both patients showed first cousin consanguinity between parents and a history of similarly affected died siblings. Moreover, one of the two patients died at the age of 3 months suggesting the association of severe ML II phenotype. In 2010, Cathey et al. reported the nonsense p.R587X mutation in a heterozygous pattern, co-occurring with a heterozygous frame-shift mutation in the same exon in USA and it was classified as a pathogenic mutation. Also, it was reported in the Brazilian population [3, 5].

Functional analysis revealed that this mutation leads to expression of C-terminally truncated α/β-subunit precursor protein. The transcript level of the nonsense mutation p.R587X mRNA was decreased by 40%, suggesting that these mutant mRNAs are unstable. This premature termination led to loss of ER exit signals in the cytoplasmic domain of the β-subunit and therefore it is retained in the ER, S1P-mediated proteolytic activation is inhibited which in turn caused the complete loss of GlcNAc-1-phosphotransferase activity. This was consistent with the severe phenotype associated with this mutation in homozygous pattern [24].

A novel frameshift mutation of 1 bp deletion in exon 19 (c.3488_3488delC; p.T1163KfsX2) was detected in homozygous pattern in a female patient who was a product of consanguineous parents. The patient presented at birth with coarse facies and skeletal deformities. Recurrent upper respiratory tract infections and delayed mental and motor milestones were the main complaints. The patient also showed brachycephaly, anteriorly bowed tibia and fibula, flared lower ribs, right side inguinal hernia, and mild atrophic brain changes were reported in the brain CT. Her clinical picture suggested a severe form of ML II. On DNA level, the C-base deletion changed threonine at position 1163 into lysine and led to a premature stop codon two amino acids away from the deletion site (p.T1163KfsX2). This change occurred in the conserved Stealth domain of β-subunit (from amino acid 1149 to 1185), which has been proposed to mediate the catalytic function of GlcNAc-1-phosphotransferase. ML II severe forms are caused by frameshift and nonsense mutations. Thus, a frameshift mutation in such important domain most probably be damaging and is responsible for the phenotype observed in our patient. According to American College of Medical Genetics and Genomics (ACMG) standards and guidelines, this variant is considered as a pathogenic variant [20].

Collectively, although clinical presentations were variable and heterogeneous among the studied group, yet they were compatible with the expected biochemical, E.M., and molecular findings. The detected increased activities of β-Hexosaminindase-A in extracellular matrices among the studied group are in agreement with the known biochemical pathogenesis of ML II. The E.M. findings in turn conformed to both clinical, biochemical, and molecular diagnosis in all patients [10].

These results suggest that the detected mutations in this study affected the assembly and function of α/β subunits in ML II patients. In turn, this led to defective activity of GlcNAc-1-phosphotransferase enzyme. β-Hexosaminindase-A is one of the affected lysosomal enzymes by this defect as it requires M6P marker for proper targeting to lysosomes. As predicted, this defect propagated to the characteristic clinical presentations and E.M picture found in the patients under study. The molecular findings were able to confirm the predicted effect of each mutation on the protein, and hence establish a promising correlation between the 4 stages of diagnosis.

The study included 8 patients, which is a reasonable sample size for such rare disorder and single-center referrals.

## Conclusions

In conclusion, 62.5% of the mutation carrying alleles (10/16 alleles) among the studied patients had the same mutation; c.3503_3504delTC (p.L1168QfsX5). This may signify the frequency of this mutation as a common disease causing variant for the ML II Egyptian patients. The high rate of parental consanguinity in our population increases the occurrence of homozygous mutations in probands. Prenatal diagnosis of ML is accurately confirmed by molecular studies in families with previously affected siblings. The quaternary diagnostics scheme of ML II should include clinical assessment, biochemical evaluation of enzymes, E.M examination of gingival inclusion bodies, and molecular study of *GNPTAB* gene.

## Recommendations

A future study on a larger group of ML II patients to determine the frequency of ML II and III among Egyptian patients is recommended. Since ML II is rare and usually misdiagnosed, employment of *GNPTAB gene* molecular analysis among the first line diagnostic battery is to be considered along with lysosomal enzyme activity assay.

## Data Availability

The datasets used and/or analyzed during the current study are available from the corresponding author on reasonable request.

## Abbreviations

ACMG: American College of Medical Genetics and Genomics
AF: Amniotic fluid
dsDNA: Double-stranded DNA
E.M.: Electron microscopy
GAGs: Glycosaminoglycans
LMSDC: Limb Malformations & Skeletal Dysplasia Clinic
ML II: Mucolipidosis type II
MPS: Mucopolysaccharidoses
mRNA: Messenger RNA
NGS: Next-generation sequencing
